# Healthcare Use for Pain in Women Waiting for Gynaecological Surgery

**DOI:** 10.1155/2016/1343568

**Published:** 2016-03-29

**Authors:** Sarah Walker, Wilma M. Hopman, Meg E. Carley, Elizabeth G. Mann, Elizabeth G. VanDenKerkhof

**Affiliations:** ^1^School of Nursing and Department of Anesthesiology and Perioperative Medicine, Queen's University, Kingston, ON, Canada K7L 3N6; ^2^Clinical Research Centre, Kingston General Hospital, Kingston, ON, Canada K7L 2V7; ^3^Department of Public Health Sciences, Queen's University, Kingston, ON, Canada K7L 3N6; ^4^School of Nursing, Queen's University, Kingston, ON, Canada K7L 3N6; ^5^Sally Smith Chair in Nursing, School of Nursing and Department of Anesthesiology and Perioperative Medicine, Queen's University, Kingston, ON, Canada K7L 3N6

## Abstract

*Background.* Pain while waiting for surgery may increase healthcare utilization (HCU) preoperatively.* Objective.* Examine the association between preoperative pain and HCU in the year prior to gynecological surgery.* Methods.* 590 women waiting for surgery in a Canadian tertiary care centre were asked to report on HCU in the year before surgery. Pain was assessed using the Brief Pain Inventory.* Results.* 33% reported moderate to severe pain intensity and interference in the week before surgery. Sixty-one percent (*n* = 360) reported a total of 2026 healthcare visits, with 21% (*n* = 126) reporting six or more visits in the year before surgery. After controlling for covariates, women with moderate to severe (>3/10) pain intensity/interference reported higher odds of overall HCU (≥3 pain-related visits to family doctor or specialist in the past year or ≥1 to emergency/walk-in clinic) compared to women with no or mild pain. Lower body mass index (BMI < 30 versus ≥30) and anxiety and/or depression were associated with emergency department or walk-in visits but not visits to family doctors or specialists.* Conclusions.* There is a high burden of pain in women awaiting gynecological surgery. Decisions about resource allocation should consider the impact of pain on individuals and the healthcare system.

## 1. Introduction

Waiting for healthcare has been identified as a common experience for today's patients [1, 2]. Specifically, there is recognition that the waiting time for surgery has increased due to the growing demand on health services [1, 3]. In a review of the literature, waiting for surgery was found to have an impact on physical, psychological, and social factors [4, 5]. For instance, waiting for surgery is an experience frequently associated with stress [6–9]. In Canada, 49–71% of individuals waiting for surgery in 2005 reported being affected by worry, stress, and anxiety [10]. Economic costs of waiting for surgery may also impact the healthcare system; prolonged waiting times may result in additional need and utilization of healthcare resources with subsequent increased costs [4, 11, 12].

Pain has been identified as a predominant symptom frequently experienced by patients awaiting gynaecological surgery [13]. Types of pain include dysmenorrhoea, premenstrual pain, and ovulatory pain, as well as other cyclic pain, which may develop into chronic pelvic pain [14]. For women waiting for gynaecological surgery, the experience of pain may also interfere with their ability to work, sleep, and enjoy social and recreational activities [13].

Although pain [15–18] and pain-related interference and disability [19–22] are recognized as contributing factors in increased healthcare utilization (HCU) and costs, evidence regarding the impact of pain on HCU for women awaiting gynaecological surgery has not been examined. A recent study describing the conditions specific to women and their ensuing HCU found that one-fifth of women have sought healthcare for female-specific conditions during a single year, with gynaecological disorders being the most commonly cited reason [23]. Zondervan and colleagues found that 59% of women who experienced pelvic pain sought medical treatment for their symptoms [24]. Also, Grace and Zondervan identified that 36% of women who had recently sought healthcare had done so because of experiencing pain [25]. Further research is needed to describe and quantify women's health issues and subsequent healthcare needs [26]. The purpose of this study is to document pain and other physical and psychological characteristics associated with HCU in women waiting for gynaecological surgery. The objectives were to describe the pain-related HCU of women waiting for gynaecological surgery, describe their health, clinical, and psychological characteristics, and explore the relationship between these characteristics and pain-related HCU.

## 2. Methods

### 2.1. Study Design

This was a single centre cross-sectional study of women waiting for a gynaecological surgical procedure. Data for this study were collected as part of a prospective study on the development of chronic postsurgical pain in women undergoing gynaecological procedures [13]. Recruitment and consent were carried out at the time of admission for surgery. The convenience sample was drawn from the waiting list of women from Kingston General Hospital, a tertiary care facility in southeastern Ontario, which serves more than 500,000 people in the local and surrounding community. Participants were asked to report recent physical and psychological symptoms and HCU for pain over the previous 12 months. This study was reviewed for ethical compliance by the Queen's University Health Sciences and Affiliated Teaching Hospitals Research Ethics Board.

### 2.2. Inclusion/Exclusion Criteria

All participants were English-speaking women aged 18 years or older waiting to undergo gynaecological surgery. Patients were excluded if they were diagnosed with Alzheimer's disease or another form of cognitive impairment such as dementia or a neurological disorder.

### 2.3. Conceptual Framework

The Theory of Unpleasant Symptoms was used to guide the methodology for the prospective study and it also applies to the current study. The theory illustrates the interplay between situational, psychological, and physiological factors and their effect on the experience of symptoms [27]. For this study, the conceptual framework was adapted to some extent. The situational factor was being on a waiting list for surgery; the psychological factors included depression and anxiety; the physiological factor was the diagnosis of a gynaecological condition; and the unpleasant symptom was pain. The theory then illustrates that the interaction of symptoms and patient characteristics leads to a performance or activity, which in this study relates to HCU for pain. The performance of HCU may have a feedback effect on the situational, psychological, and physiological factors, and it is also postulated within this conceptual framework that the feedback loop could affect performance.

### 2.4. Measures

The independent variables were waiting time (situational), depression and anxiety (psychological), gynaecological diagnosis (physiological), and pain (physical symptom). As pain is often a major component of HCU, the primary dependent variable for this study was pain-related HCU. Covariates included demographic, surgical, and gynaecological factors.

### 2.5. Measurement Tools

Data collection consisted of 5 self-completed questionnaires capturing information on pain, psychological factors, and HCU. Additional clinical data on smoking status, body mass index (BMI), and registration with a family practitioner were gathered from reviewing the patient record.

### 2.6. Demographic and Clinical Covariates

Potential covariates included demographic and clinical variables that are potentially or known to be associated with the primary outcome of HCU. Age was examined as a continuous variable and categorized according to the documented menopausal range of 45–55 years [28], creating the groups of premenopausal (18–44 years), menopausal (45–55 years), and postmenopausal (≥56 years). Marital status was categorized as married or not married. Due to the small size of some of the racial categories, the variable of racial heritage was divided into Caucasian and non-Caucasian groups. Education was classified into four groups including no diploma, high school diploma, trade or professional school certificate/diploma, and some university/postgraduate degree(s). Employment was classified into 3 groups: (i) part time or full time, (ii) not employed, retired, or homemaker, and (iii) other. BMI was categorized according to WHO classification, with the ranges of underweight/normal weight ≤ 24.9 kg/m^2^, overweight 25–29.9 kg/m^2^, and obese ≥ 30 kg/m^2^ [29]. The underweight and normal categories were combined due to the small number of participants in the underweight, <18.5 kg/m^2^, range. Participants were also classified by current smoking status and by whether they had undergone previous abdominal surgery.

### 2.7. Situational Factors

The participants enrolled in this study all had a period of waiting time for surgery and these data were obtained from administrative data captured by the hospital. “Wait 2” is defined as the time between the decision to treat and the date of surgery. For this analysis the “adjusted days waited” was used as it adjusts for any time individuals may not have been available for surgery (e.g., vacation).

### 2.8. Psychological Factors

Trait anxiety was measured using the State Trait Anxiety Inventory (STAI), a self-report measurement tool of 20 questions asking about general feelings. The ranges of scores were 20–80 with a higher score indicating a greater degree of anxiety. The STAI was used by Carr et al. to examine anxiety in women prior to and following gynaecological surgery [30] and by Oudhoff et al. to examine patients waiting for surgery [9]. For this study, participants were categorized based on the trait anxiety score of <45 (low anxiety), or ≥45 (high anxiety) [30].

Depression was measured using the Centre for Epidemiologic Studies-Depression (CES-D) Scale, a 20-item questionnaire designed to study depression in the general population. It involves self-reporting of feelings during the past week. The scoring for depression was the total out of 60 possible points, with a higher score indicating a greater degree of depression. This tool has been used extensively to study chronic pain and depression [31–33]. For this study, participant categories were based on a CES-D score < 16 or ≥16 or more which indicates a greater number of depressive symptoms and suggests a risk of depression that requires treatment [34–36].

A variable consisting of both trait anxiety and depression was also created, due to the high coexistence of anxiety and depression. Individuals were classified as having no depression (<16/60) or anxiety (<45/80) or as having one or both.

### 2.9. Physiological Factors

Self-reported menstrual status was classified into no longer menstruating due to natural or surgical means or not stopped/unsure. An assumption was made that if they were unsure about their periods stopping, some recent bleeding had probably occurred, and these participants were assigned to the “not stopped/unsure” category. Data regarding hormone replacement therapy (HRT) and oral contraceptives were collected as possible factors related to hormones and the pain experience; in each case, the participants were grouped according to whether they reported taking the medication or not. Lastly, self-reported preoperative malignancy status was classified as possibly malignant, malignant, or not malignant. While a confirmed diagnosis was available from hospital administrative data postoperatively, we felt the preoperative self-reported status was most relevant to HCU while waiting for surgery.

### 2.10. Physical Symptom (Pain)

The Brief Pain Inventory Long Form (BPI-LF), a multidimensional assessment instrument, measures the severity of pain and the impact of pain on daily function [37]. Participants rate the intensity of their pain on four scales using numerical scales of 0 (no pain) to 10 (pain as bad as you can imagine). Pain interference is rated for seven activities during the past week between 0 (does not interfere) and 10 (completely interferes). An average pain intensity score was computed using the four pain intensity variables from the BPI-LF (worst pain over the past week, least pain over the past week, pain on average, and pain “right now”), while an average pain interference score was computed using seven interference variables from the BPI-LF (general activity, mood, walking ability, normal work, relations, sleep, and enjoyment of life). Pain was dichotomized into what is considered to be a clinically relevant cut-off, none/mild (≤3/10) and moderate/severe (>3/10) [38, 39].

### 2.11. Performance (Primary Outcome), HCU

Pain-related HCU was captured using questions adapted from the Canadian National Population Health Survey. Individuals were asked to report on the number of visits to a family doctor, specialist, walk-in clinic, emergency department (ED), or other healthcare professional “because of pain” in the past 12 months.* Planned* HCU (i.e., family doctor, specialist) was dichotomized into high (≥3 visits) versus low (<3) [40]. The rationale for <3 visits to be considered low was that an individual may have one scheduled visit to a family doctor for a check-up and possibly an additional visit due to the pending surgery. Urgent or emergent HCU (i.e., walk-in or ED) is usually unplanned HCU and therefore any visit to ED or an urgent care setting (i.e., >0) was considered as high use. Results from studies using self-report questions about HCU have been reported. Barsky et al. used a self-report questionnaire when examining HCU patterns of patients who somatise [17], and Patel et al. reported good accuracy with recall compared to chart review [41].

### 2.12. Statistical Analysis

Descriptive statistics were calculated using frequency and percentage for categorical variables and mean, standard deviation, range, and interquartile range for continuous variables. Bivariate analysis was conducted to examine factors associated with HCU using the Chi square statistical test for categorical variables and *t*-test for continuous variables. Two-way interactions were conducted to assess how pain interacts with different classes of variables to influence healthcare use. If *p* < 0.10 for an interaction term it was included in the multivariable logistic regression analysis. The interaction term was removed from the full model if *p* > 0.05. Statistically significant interaction terms were further explored through stratified analysis. Finally, multivariable modelling was guided by the conceptual framework whereby a block of variables (e.g., psychological) was entered into the multiple logistic regression analysis and variables were removed one at a time until only those with *p* < 0.10 remained. A *p* < 0.10 was used to avoid missing variables that were close to being statistically significant. The unadjusted, fully adjusted, and reduced adjusted models are provided. Due to the high percent agreement between pain intensity and pain interference, separate logistic regression models were created for these variables. Therefore, a total of 6 multivariable analyses were conducted: two for each of the 3 HCU outcomes (family doctor, specialist, and ED or walk-in), one with pain intensity as the primary “exposure” of interest, and one with pain interference as the primary “exposure” variable. All analyses were carried out using the IBM® SPSS® software version 22.0. Given the descriptive nature of this study, a sample size calculation was not conducted.

## 3. Results

**Figure 1:**
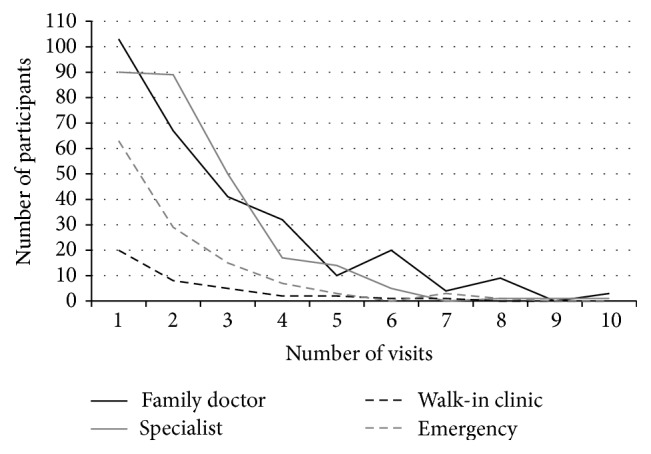
Number of healthcare visits for pain during 12 months prior to surgery. Number of participants with no visits in previous year: family doctor = 283, specialist = 317, walk-in clinic = 548, and emergency = 468. Number of participants with more than 10 visits in previous year (included in the group with 10 visits): family doctor = 15, specialist = 3, and walk-in clinic = 2.

Of the 932 participants approached and invited to join the study, 696 (74.7%) agreed to participate and 590 (63.3%) completed the questionnaires. Sixty-eight percent (67.8%) of the participants were scheduled for a hysterectomy and 23.6% were scheduled for other uterine, tubal, or ovarian procedures. The remaining 8.6% were scheduled for exploratory, vulvar, or pelvic floor procedures. Of the 590 participants, 360 (61.3%) reported a total of 2026 healthcare visits to a family doctor, specialist, ED, or walk-in clinic for pain in the previous 12 months (Figure 1). This represents an average of 3.5 (median 2.0) visits per person and for the 360 who had at least 1 pain-related visit, the average was 5.6 (median 4.0) visits per person. The majority (51.8%) sought care from the family doctor, followed by a specialist (46.1%), an ED (20.5%), and a walk-in clinic (7.0%). Thirty percent of participants visited other healthcare professionals over the previous 12 months, with a mean of 3.9 visits and a range of 0–156. Frequency of visits to family doctor or specialist was highly correlated with frequency of visits to EDs or walk-in clinics. For example, 36.6% (*n* = 49) of participants who frequently visited the family doctor also visited ED or walk-in clinic, while only 3.2% (*n* = 9) of participants who did not see family doctor visited ED or walk-in clinic.

Situational, psychological, physiological, pain, clinical, and demographic characteristics for the total sample (*n* = 590) are provided in Table 1. Age of the study sample ranged from 18 to 86 years with a mean of 48.3 (standard deviation [SD] = 11.3). Adjusted days waiting for surgery ranged from 0 to 645 days, with a median of 46 days (25th/75th percentiles = 26/83). The mean trait anxiety score was 34.7 (SD = 10.9) out of a possible range of 20–80. The mean CES-D score was 13.7 (SD = 11.5). One-third of participants reported moderate to severe (>3/10) pain intensity over the past week and 32.8% reported moderate to severe pain interference (>3/10). Examination of individual interference scale items revealed that 44.9% (*n* = 264) of women reported moderate to severe interference on at least one item and approximately one-third reported moderate to severe interference with sleep (*n* = 194, 33.0%), general activity (*n* = 190, 32.3%), enjoyment with life (*n* = 188, 32.0%), mood (*n* = 187, 31.8%), and normal work (*n* = 182, 31.0%). Twenty-six percent (*n* = 151, 25.7%) reported moderate to severe interference with walking ability and 20.4% (*n* = 120) reported moderate to severe interference with relations. Of the participants that experienced pain in the last week, 75.2% believed their pain was due to their primary gynaecological condition.

### 3.1. Relationship between Symptoms and HCU for Pain

Potentially significant (*p* < 0.10) relationships existed between pain intensity and each of the following independent variables: age, race, BMI, smoking, anxiety, and depression. Significant relationships existed between pain interference and age, BMI, anxiety, depression, and menstrual status. Interactions terms for these relationships were entered into the relevant multivariable logistic regression analyses. The interaction between pain and age in the family doctor model was the only interaction that remained significant in the full model.

Generally, the pain intensity and pain interference models were similar (Tables 2 [Table tab3]–4). The family doctor and specialist outcomes were also similar. In the reduced model, pain, younger age, currently smoking, previous abdominal surgery, and nonmalignancy were associated with 3 or more healthcare visits to the family doctor or a specialist in the year before surgery (Tables 2 and 3). Being married also increased the odds of going to the family doctor. However, with the exception of age and pain, these same factors were not associated with seeking care from an ED or walk-in clinic (Table 4). Instead, BMI < 30 (OR = 1.44, 95% CI 0.94–2.22, reference BMI ≥ 30) and anxiety and/or depression (OR = 1.54, 95% CI 0.99–2.38, with reference being neither depression nor anxiety) were associated with ED or walk-in visits.

In the adjusted analysis where age and pain intensity/interference were stratified for visits to the family doctor (Table 5), women under the age of 50 and with moderate to severe pain intensity had significantly greater odds of seeking frequent care from the family doctor than women under the age of 50 with no or mild pain (OR = 3.46, 95% CI 2.11–5.69). Women over the age of 50 with no or mild pain had the lowest odds of seeking frequent healthcare (OR = 0.30, 95% CI 0.14–0.65). Similar findings were present for age and pain interference.

## 4. Discussion

The overall aim of this study was to gain a better understanding of pain-related healthcare needs of participants waiting for gynaecological surgery. Approximately one-third of these women experienced symptoms of mental distress, pain interference, and/or moderate to severe pain intensity during the preoperative period. One of the major findings was that the average participant sought healthcare for pain 3.5 times in the 12 months before surgery, and family doctors were the main settings from which they sought help; however, frequent visits to the family doctor did not necessarily mean fewer visits to the ED. In addition, different characteristics were associated with seeking care from EDs or walk-in clinics, compared to the family doctor or a specialist. Depression and/or anxiety, which is highly correlated with pain, was associated with visits to an ED or walk-in clinic but not the family doctor or a specialist.

Overall, our results show that 20% of participants experienced symptoms of anxiety and 37% demonstrated a risk of depression needing treatment, which are similar to other reports of preoperative mental distress in women waiting for gynaecological surgery [30, 42–47]. Longitudinal studies have identified the preoperative period as being the most mentally distressing, with scores for both depression and anxiety decreasing postoperatively [42, 45–47]. As both depression [19, 21, 22, 48, 49] and anxiety [48, 50] have been associated with increased healthcare use in other pain groups, it is not surprising that women with high anxiety and/or depression in this study were more likely to utilize healthcare for pain when compared to participants with lower anxiety or depression scores. However, we only found this to be true for urgent/emergent care and not for what might be considered planned appointments to the family doctor or specialist. As the preoperative period has been identified as the time when anxiety and depression peak [42, 45–47], screening patients for anxiety and depression during the preoperative assessment may help in identifying who may require additional interventions to decrease the mental strain of the preoperative period and potentially reduce additional pain-related HCU in particular in the ED and walk-in clinics.

Approximately one-third of the study participants experienced pain of at least a moderate intensity and level of interference, suggesting that there is a significant burden of pain experienced in women waiting for gynaecological surgery. As over 60% of participants reported at least one pain-related healthcare visit in the preceding year, these estimates of pain intensity and interference are likely conservative. Participants experiencing severe interference with normal work activities only, for example, may have had a low total interference score yet still visited a health professional for care. Our finding that participants with moderate to severe pain intensity and pain interference were more likely to seek healthcare is consistent with prior research in pain groups [19, 21, 22, 51–53].

Having a history of prior abdominal surgery was a predictor of high pain-related HCU. No other studies have examined or identified the association between history of surgery and HCU. Individuals with prior abdominal surgery may have more complex health conditions, including chronic postsurgical pain [13, 54, 55], than those without a history of prior surgery, which may result in higher HCU for pain.

### 4.1. Study Limitations

Study limitations include the use of self-report of HCU and physiological and psychological symptoms. Recall bias may be present due to the need to recall HCU for pain over the previous 12 months. A review of literature on recall bias regarding HCU found conflicting information between the accuracy of patient self-reports and medical records. The underreporting of HCU was found in cases where HCU is high [56, 57], and individuals with poor health status were found to over-report HCU [57]. In this study, selection bias may have led to an overestimation of HCU if patients with higher levels of pain were more likely to over-report HCU compared to those with no or mild pain. Participants were asked to report on HCU specifically for pain. Participants may have underestimated the number of pain-related visits if pain coincided with other conditions or symptoms. However, it is well documented that HCU is frequently related to pain, with up to 80% of all visits to physicians having a pain-related component [58]. It should be noted that this study collected HCU in the year prior to surgery, so it remains unknown what health resource needs women have specific to the period between a decision to proceed with surgery and actually having the surgery. While women were asked to report on any pain-related healthcare use, the exact reason for the visit was not captured. Given that pain is a subjective experience, the reason for the visit could be considered to be of less importance than the fact that women may be suffering from pain unnecessarily. However, these results cannot be interpreted to mean that the pain was related to the reason for gynaecologic surgery.

The primary purpose of the main study was to understand the development of chronic postsurgical pain [13]. It is unknown to what extent the participants who enrolled were more interested because of a personal experience with pain. On the contrary, participants with high levels of pain may not have felt well enough to complete the questionnaires prior to surgery. Therefore, it is not known whether selection bias might affect the study. However, the finding that 55% of the sample experienced pain in the past week is in line with a pain prevalence study conducted in the same region where 60% of the sample reported some degree of pain [53].

### 4.2. Study Strengths

The response rate of 63% is higher than rates from prevalence studies on chronic pain [53, 59, 60] and higher than the declining rates found in many epidemiologic studies [61–63]. The sample size of 590 participants was sufficient to allow several independent variables to be included in the regression. Furthermore, the comprehensive data collected, situational, psychological, physiological, and demographic factors, is unique to the literature on studies examining factors related to the experience of waiting for surgery. A further strength of this study is the fact that the recall period for reporting factors and symptoms was short therefore diminishing the chance of recall bias.

### 4.3. Implications for Healthcare Professionals

The results suggest that individuals with a history of prior abdominal surgery and either moderate to severe pain intensity or interference would make good targets for interventions to reduce high HCU. As primary care was the most commonly identified setting from which women sought pain-related care, this may be the ideal setting to deliver interventions. There is currently a paucity of interventions that have evaluated the effect of pelvic pain interventions on HCU [64]; however, there is evidence to support HCU interventions in other pain groups including self-management [65] and nurse-led pain management clinics [66]. As many of these interventions may require longer than the preoperative period identified in this study (median: 46 days), interventions to reduce pain-related HCU may be most effective if delivered when women initially present to primary care with gynecologic pain issues. Multiple low-cost interventions have been developed to reduce pain-related ED visits making it feasible to deliver these interventions to a large number of individuals. Examples of these interventions include single behavioural health or psychoeducation interventions delivered over 15–30 minutes in the ED [67].

Empirical evidence suggests that management of pain improves with early intervention [68]. Healthcare professionals may feel limited in their ability to assist patients in managing their pain [69]; however, this study provides an improved understanding of the impact of unpleasant symptoms, such as anxiety and pain, which patients experience while waiting for surgery. Waddell suggests that efficiency and planning of surgical procedures from the preoperative stage will lead to better-quality patient education and improved patient expectations and potentially improve satisfaction with the experience [70]. Transparent and efficient presurgical processes may facilitate health promotion, improve patient satisfaction, and subsequently improve cost-effectiveness through reduced HCU for pain, despite waiting times. This may reduce the burden on the patient and on primary care and may indirectly reduce the need for patients to attend the ED or walk-in clinic for pain. A recommendation could be to implement preoperative care pathway planning that involves addressing pain management for patients undergoing gynaecological surgery with the aim of reducing the impact waiting for surgery has on both the patient and the healthcare system.

### 4.4. Summary

This study has provided evidence that women experience unpleasant symptoms while waiting for gynaecologic surgery. In particular, a substantial number of visits to healthcare providers occur because of pain. Consideration of physical and psychological factors, in addition to issues directly related to the primary reason for surgery, may improve outcomes while reducing HCU at the same time as individuals await surgery. However, further research is needed to assess the effectiveness of interventions designed to target psychological and physical needs of patients prior to surgery.

## Figures and Tables

**Table 1:** Baseline characteristics of women waiting for gynaecological surgery.

	Total (*n* = 590) *n* (%)^*∗*^
Demographic covariates	
Age^†^	
Years (mean (SD))	48.3 (11.3)
Age^†^	
18–44 years	227 (38.9)
45–55 years	213 (36.5)
≥56 years	144 (24.7)
Marital status^‡^	
Single/divorced/widowed	167 (28.4)
Married	422 (71.6)
Racial heritage^§^	
Caucasian	540 (92.9)
Non-Caucasian	41 (7.1)
Highest education grade achieved^‡^	
No diploma	69 (11.7)
High school diploma	118 (20.0)
Trade or professional school certificate/diploma	221 (37.5)
Some university/postgraduate	181 (30.7)
Employment status^||^	
Unemployed/retired/homemaker	155 (26.4)
Employed part time or full time	384 (65.3)
Other	49 (8.3)

Clinical covariates	
Body mass index (kg/m^2^)^†^	
Underweight/normal (≤24.9)	155 (26.5)
Overweight (25–29.9)	175 (30.0)
Obese (≥30)	254 (43.5)
Current smoker^‡^	
Yes	123 (20.9)
No	466 (79.1)
Previous abdominal surgery^†^	
Yes	404 (69.2)
No	180 (30.8)

Situational factors	
Adjusted days waited^||^	
<4 weeks	155 (26.4)
4–8 weeks	198 (33.7)
>8 weeks	235 (40.0)

Psychological factors	
Trait anxiety score^¶^	
<45	469 (80.4)
≥45	114 (19.6)
CES-D score^||^	
<16	372 (63.3)
≥16	216 (36.7)
Anxiety and/or depression	
No	353 (60.5)
Yes	230 (39.5)

Physiological factors, gynaecological	
Current menstruation status	
Not stopped	305 (51.7)
Unsure/irregular	42 (7.1)
Stopped naturally	181 (30.7)
Stopped surgically	62 (10.5)
Taking hormone replacement therapy	
Yes	34 (5.8)
No	556 (94.2)
Birth control pills in the past month^*∗∗*^	
Yes	46 (7.9)
No	536 (92.1)
Preoperative malignancy status^||^	
Possibly malignant	137 (23.3)
Malignant	102 (17.3)
Not malignant	349 (59.4)

Physical symptoms	
Pain intensity scale (BPI)^||^	
≤3/10	392 (66.7)
>3/10	196 (33.3)
Pain interference scale (BPI)^||^	
≤3/10	395 (67.2)
>3/10	193 (32.8)

^*∗*^Values do not always equal 100% due to rounding; ^†^6 participants missing; ^‡^1 participant missing; ^§^9 participants missing; ^||^2 participants missing; ^¶^7 participants missing; ^*∗∗*^8 participants missing; BPI: Brief Pain Inventory; CES-D: Centre for Epidemiological Studies-Depression.

**Table 2:** Unadjusted and adjusted analysis of association between *pain intensity/pain interference* and ≥3 visits to the family doctor for pain in the past 12 months.

	<3 visits	≥3 visits	Pain intensity model OR (95% CI)			Pain interference model OR (95% CI)		
	*n* (%)	*n* (%)	Unadjusted	Adjusted (full)	Adjusted (reduced)	Unadjusted	Adjusted (full)	Adjusted (reduced)
Demographic covariates								
Age *∗* pain interaction term			1.07 (1.02–1.11)	1.09 (1.04–1.14)	1.08 (1.03–1.13)	1.08 (1.04–1.12)	1.10 (1.05–1.15)	1.10 (1.05–1.14)
Marital status								
Single/divorced/widowed	133 (80.1)	33 (19.9)	1.0	1.0	1.0	1.0	1.0	1.0
Married	320 (76.2)	100 (23.8)	1.26 (0.81–1.96)	1.58 (0.93–2.67)	1.35 (0.82–2.22)	1.26 (0.81–1.96)	1.59 (0.94–2.70)	1.38 (0.84–2.27)
Employment status								
Not employed/retired/other	164 (80.4)	40 (19.6)	1.0	1.0	NS	1.0	1.0	NS
Employed full time or part time	287 (75.3)	94 (24.7)	1.34 (0.89–2.04)	1.26 (0.75–2.12)		1.34 (0.89–2.04)	1.30 (0.77–2.18)	
Education								
High school diploma or less	146 (78.5)	40 (21.5)	1.0	1.0	NS	1.0	1.0	NS
More than high school	307 (76.8)	93 (23.3)	1.11 (0.73–1.68)	1.08 (0.65–1.80)		1.11 (0.73–1.68)	1.01 (0.61–1.67)	

Clinical covariates								
BMI								
≥30	196 (77.2)	58 (22.8)	1.0	1.0	NS	1.0	1.0	NS
<30	252 (77.1)	75 (22.9)	1.01 (0.68–1.49)	1.10 (0.69–1.74)		1.01 (0.68–1.49)	1.19 (0.75–1.88)	
Current smoker								
No	369 (79.5)	95 (20.5)	1.0	1.0	1.0	1.0	1.0	1.0
Yes	84 (68.9)	38 (31.1)	1.76 (1.13–2.74)	1.41 (0.85–2.37)	1.41 (0.86–2.32)	1.76 (1.13–2.74)	1.50 (0.90–2.50)	1.46 (0.89–2.40)
Previous abdominal surgery								
No	154 (86.0)	25 (14.0)	1.0	1.0	1.0	1.0	1.0	1.0
Yes	296 (73.6)	106 (26.4)	2.21 (1.37–3.56)	2.37 (1.37–4.09)	2.16 (1.28–3.65)	2.21 (1.37–3.56)	2.45 (1.41–4.24)	2.31 (1.37–3.91)

Situational factors								
Adjusted days waited								
≤8 weeks	272 (77.5)	79 (22.5)	1.0	1.0	NS	1.0	1.0	NS
>8 weeks	180 (76.9)	54 (23.1)	1.03 (0.70–1.53)	0.75 (0.47–1.22)		1.03 (0.70–1.53)	0.81 (0.50–1.30)	

Psychological factors								
Anxiety and/or depression								
No	287 (81.8)	64 (18.2)	1.0	1.0	NS	1.0	1.0	NS
Yes	161 (70.0)	69 (30.0)	1.92 (1.30–2.84)	1.10 (0.69–1.75)		1.92 (1.30–2.84)	1.01 (0.63–1.63)	

Physiological factors, gynaecological								
Current menstruation status								
Not stopped or unsure	258 (74.8)	87 (25.2)	1.0	1.0	NS	1.0	1.0	NS
Stopped surgically or naturally	195 (80.6)	47 (19.4)	0.71 (0.48–1.07)	1.35 (0.73–2.48)		0.71 (0.48–1.07)	1.49 (0.81–2.73)	
Taking hormone replacement therapy								
No	431 (77.8)	123 (22.2)	1.0	1.0	NS	1.0	1.0	NS
Yes	22 (66.7)	11 (33.3)	1.75 (0.83–3.71)	1.75 (0.73–4.21)		1.75 (0.83–3.71)	1.76 (0.73–4.24)	
Birth control pills in the past month								
No	415 (77.9)	118 (22.1)	1.0	1.0	NS	1.0	1.0	NS
Yes	33 (71.7)	13 (28.3)	1.39 (0.71–2.72)	1.13 (0.52–2.48)		1.39 (0.71–2.72)	1.22 (0.55–2.67)	
Preoperative malignancy status								
Possibly/definitely malignant	198 (83.2)	40 (16.8)	1.0	1.0	1.0	1.0	1.0	1.0
Not malignant	254 (73.2)	93 (26.8)	1.81 (1.20–2.74)	1.42 (0.83–2.43)	1.44 (0.89–2.33)	1.81 (1.20–2.74)	1.44 (0.85–2.45)	1.45 (0.90–2.34)

Note: main effects for the interaction term age *∗* pain were included in the full and reduced adjusted models; age is as a continuous variable and pain scales (intensity and interference) were categorized at ≤3 or >3.

The pain intensity model included the age *∗* pain interference interaction term. The pain interference model included the age *∗* pain intensity interaction.

BPI: Brief Pain Inventory, NA: not applicable, and NS: not selected for final model.

**Table 3 tab3:** Unadjusted and adjusted analysis of association between *pain intensity/pain interference* and ≥3 visits to a specialist for pain in the past 12 months.

	<3 visits	≥3 visits	Pain intensity model OR (95% CI)			Pain interference model OR (95% CI)		
	*n* (%)	*n* (%)	Unadjusted	Adjusted (full)	Adjusted (reduced)	Unadjusted	Adjusted (full)
Physical symptoms								
Pain intensity scale (BPI)								
≤3/10	355 (91.0)	35 (9.0)	1.0	1.0	1.0	NA	NA	NA
>3/10	139 (70.9)	57 (29.1)	4.16 (2.61–6.62)	3.34 (2.00–5.56)	3.53 (2.18–5.74)	NA	NA	NA
Pain interference scale (BPI)								
≤3/10	354 (90.1)	39 (9.9)	NA	NA	NA	1.0	1.0	1.0
>3/10	140 (72.5)	53 (27.5)	NA	NA	NA	3.44 (2.17–5.43)	2.88 (1.69–4.91)	2.90 (1.79–4.69)

Demographic covariates								
Age								
Years (mean (SD))	49.2 (11.4)	44.0 (9.6)	0.96 (0.94–0.98)	0.96 (0.93–1.00)	0.97 (0.95–0.99)	0.96 (0.94–0.98)	0.96 (0.93–0.99)	0.97 (0.95–1.00)
Marital status								
Single/divorced/widowed	144 (86.2)	23 (13.8)	1.0	1.0	NS	1.0	1.0	NS
Married	352 (83.8)	68 (16.2)	1.21 (0.73–2.02)	1.40 (0.79–2.49)		1.21 (0.73–2.02)	1.37 (0.78–2.43)	
Employment status								
Not employed/retired/other	175 (85.8)	29 (14.2)	1.0	1.0	NS	1.0	1.0	NS
Employed full or part time	319 (83.5)	63 (16.5)	1.19 (0.74–1.92)	1.03 (0.59–1.80)		1.19 (0.74–1.92)	1.03 (0.59–1.80)	
Education								
High school diploma or less	156 (83.9)	30 (16.1)	1.0	1.0	NS	1.0	1.0	NS
More than high school	339 (84.5)	62 (15.5)	0.95 (0.59–1.53)	0.91 (0.52–1.58)		0.95 (0.59–1.53)	0.89 (0.51–1.53)	

Clinical covariates								
BMI								
≥30	213 (83.9)	41 (16.1)	1.0	1.0	NS	1.0	1.0	NS
<30	278 (84.8)	50 (15.2)	0.93 (0.60–1.47)	0.97 (0.59–1.61)		0.93 (0.60–1.47)	1.00 (0.61–1.66)	
Current smoker								
No	402 (86.6)	62 (13.4)	1.0	1.0	1.0	1.0	1.0	1.0
Yes	94 (76.4)	29 (23.6)	2.00 (1.22–3.28)	1.63 (0.94–2.83)	1.70 (0.99–2.90)	2.00 (1.22–3.28)	1.72 (1.00–2.98)	1.77 (1.04–3.00)
Previous abdominal surgery								
No	161 (89.9)	18 (10.1)	1.0	1.0	1.0	1.0	1.0	1.0
Yes	332 (82.4)	71 (17.6)	1.91 (1.10–3.32)	1.73 (0.94–3.15)	1.69 (0.95–3.00)	1.91 (1.10–3.32)	1.74 (0.95–3.17)	1.72 (0.97–3.04)

Situational factors								
Adjusted days waited								
≤8 weeks	300 (85.2)	52 (14.8)	1.0	1.0	NS	1.0	1.0	NS
>8 weeks	194 (82.9)	40 (17.1)	1.19 (0.76–1.87)	0.91 (0.54–1.54)		1.19 (0.76–1.87)	0.96 (0.57–1.62)	

Psychological factors								
Anxiety and/or depression								
No	309 (87.8)	43 (12.2)	1.0	1.0	NS	1.0	1.0	NS
Yes	181 (78.7)	49 (21.3)	1.95 (1.24–3.05)	1.21 (0.73–2.02)		1.95 (1.24–3.05)	1.13 (0.67–1.91)	

Physiological factors, gynaecological								
Current menstruation status								
Not stopped or unsure	284 (82.1)	62 (17.9)	1.0	1.0	NS	1.0	1.0	NS
Stopped surgically or naturally	212 (87.6)	30 (12.4)	0.65 (0.40–1.04)	1.19 (0.61–2.32)		0.65 (0.40–1.04)	1.29 (0.66–2.52)	
Taking hormone replacement therapy								
No	472 (85.0)	83 (15.0)	1.0	1.0	NS	1.0	1.0	NS
Yes	24 (72.7)	9 (27.3)	2.13 (0.96–4.75)	2.14 (0.88–5.19)		2.13 (0.96–4.75)	2.12 (0.88–5.12)	
Birth control pills in the past month								
No	450 (84.3)	84 (15.7)	1.0	1.0	NS	1.0	1.0	NS
Yes	38 (82.6)	8 (17.4)	1.13 (0.51–2.50)	0.90 (0.36–2.24)		1.13 (0.51–2.50)	0.98 (0.39–2.44)	
Preoperative malignancy status								
Possibly/definitely malignant	214 (89.5)	25 (10.5)	1.0	1.0	1.0	1.0	1.0	1.0
Not malignant	280 (80.7)	67 (19.3)	2.05 (1.25–3.35)	1.56 (0.90–2.68)	1.56 (0.97–2.82)	2.05 (1.25–3.35)	1.60 (0.89–2.88)	1.61 (0.94–2.76)

BPI: Brief Pain Inventory, NA: not applicable, and NS: not selected for final model.

**Table 4:** Unadjusted and adjusted analysis of association between *pain intensity/interference* and ≥1 visit to a walk-in clinic or emergency department for pain in the past 12 months.

	0 visits	≥1 visit	Pain intensity model OR (95% CI)			Pain interference model OR (95% CI)		
	*n* (%)	*n* (%)	Unadjusted	Adjusted (full)	Adjusted (reduced)	Unadjusted	Adjusted (full)
Physical symptoms								
Pain intensity scale (BPI)								
≤3/10	323 (82.6)	68 (17.4)	1.0	1.0	1.0	NA	NA	NA
>3/10	123 (62.8)	73 (37.2)	2.82 (1.91–4.16)	2.48 (1.59–3.88)	2.35 (1.53–3.61)	NA	NA	NA
Pain interference scale (BPI)								
≤3/10	329 (83.5)	65 (16.5)	NA	NA	NA	1.0	1.0	1.0
>3/10	117 (60.6)	76 (39.4)	NA	NA	NA	3.29 (2.22–4.87)	3.01 (1.88–4.83)	2.81 (1.80–4.40)

Demographic covariates								
Age								
Years (mean (SD))	49.7 (11.5)	44.3 (9.4)	0.95 (0.94–0.97)	0.95 (0.93–0.98)	0.96 (0.94–0.98)	0.95 (0.94–0.97)	0.95 (0.93–0.98)	0.96 (0.94–0.98)
Marital status								
Single/divorced/widowed	130 (77.8)	37 (22.2)	1.0	1.0	NS	1.0	1.0	NS
Married	317 (75.3)	104 (24.7)	1.15 (0.75–1.77)	1.25 (0.77–2.05)		1.15 (0.75–1.77)	1.25 (0.77–2.05)	
Employment status								
Not employed/retired/other	166 (81.4)	38 (18.6)	1.0	1.0	NS	1.0	1.0	NS
Employed full time or part time	280 (73.1)	103 (26.9)	1.61 (1.06–2.44)	1.82 (1.10–3.02)		1.61 (1.06–2.44)	1.85 (1.11–3.07)	
Education								
High school diploma or less	149 (79.7)	38 (20.3)	1.0	1.0	NS	1.0	1.0	NS
More than high school	298 (74.3)	103 (25.7)	1.36 (0.89–2.06)	1.24 (0.76–2.04)		1.36 (0.89–2.06)	1.22 (0.74–2.01)	

Clinical covariates								
BMI								
≥30	201 (79.1)	53 (20.9)	1.0	1.0	1.0	1.0	1.0	1.0
<30	246 (74.8)	83 (25.2)	1.28 (0.86–1.89)	1.36 (0.87–2.11)	1.34 (0.87–2.04)	1.28 (0.86–1.89)	1.48 (0.94–2.32)	1.44 (0.94–2.22)
Current smoker								
No	362 (77.8)	103 (22.2)	1.0	1.0	NS	1.0	1.0	NS
Yes	86 (69.9)	37 (30.1)	1.51 (0.97–2.36)	1.08 (0.65–1.80)		1.51 (0.97–2.36)	1.14 (0.68–1.90)	
Previous abdominal surgery								
No	144 (80.4)	35 (19.6)	1.0	1.0	NS	1.0	1.0	NS
Yes	301 (74.5)	103 (25.5)	1.41 (0.91–2.17)	1.40 (0.86–2.28)		1.41 (0.91–2.17)	1.40 (0.85–2.29)	

Situational factors								
Adjusted days waited								
≤8 weeks	261 (73.9)	92 (26.1)	1.0	1.0	1.0	1.0	1.0	1.0
>8 weeks	186 (79.5)	48 (20.5)	0.73 (0.49–1.09)	0.54 (0.33–0.86)	0.63 (0.41–0.97)	0.73 (0.49–1.09)	0.56 (0.35–0.90)	0.65 (0.42–1.00)

Psychological factors								
Anxiety and/or depression								
No	290 (82.2)	63 (17.8)	1.0	1.0	1.0	1.0	1.0	1.0
Yes	154 (67.0)	76 (33.0)	2.27 (1.54–3.34)	2.00 (1.29–3.11)	1.75 (1.15–2.68)	2.27 (1.54–3.34)	1.74 (1.10–2.74)	1.54 (0.99–2.38)

Physiological factors, gynaecological								
Current menstruation status								
Not stopped or unsure	255 (73.7)	91 (26.3)	1.0	1.0	NS	1.0	1.0	NS
Stopped surgically or naturally	193 (79.4)	50 (20.6)	0.73 (0.49–1.07)	1.74 (0.98–3.08)		0.73 (0.49–1.07)	1.92 (1.07–3.43)	
Taking hormone replacement therapy								
No	424 (76.4)	131 (23.6)	1.0	1.0	NS	1.0	1.0	NS
Yes	24 (70.6)	10 (29.4)	1.35 (0.63–2.89)	1.39 (0.60–3.22)		1.35 (0.63–2.89)	1.34 (0.57–3.14)	
Birth control pills in the past month								
No	409 (76.4)	126 (23.6)	1.0	1.0	NS	1.0	1.0	NS
Yes	32 (69.6)	14 (30.4)	1.42 (0.73–2.74)	1.01 (0.47–2.19)		1.42 (0.73–2.74)	1.15 (0.53–2.51)	
Preoperative malignancy status								
Possibly/definitely malignant	188 (78.7)	51 (21.3)	1.0	1.0	NS	1.0	1.0	NS
Not malignant	259 (74.4)	89 (25.6)	1.27 (0.86–1.87)	1.04 (0.63–1.70)		1.27 (0.86–1.87)	1.06 (0.64–1.74)	

BPI: Brief Pain Inventory, NA: not applicable, and NS: not selected for final model.

**Table 5:** Exploration of the interaction between age and pain intensity and frequent visits to the family doctor.

≥3 visits to the family doctor					
Age	Pain intensity	<3 visits	≥3 visits	OR (95% CI)	
		*n* (%)	*n* (%)	Unadjusted	Adjusted (reduced)

≤50	≤3	185 (81.9)	41 (18.1)	1.0	1.0
≤50	>3	74 (53.2)	65 (46.8)	3.96 (2.47–6.37)	3.46 (2.11–5.69)
>50	≤3	151 (94.4)	9 (5.6)	0.27 (0.13–0.57)	0.30 (0.14–0.65)
>50	>3	38 (69.1)	17 (30.9)	2.02 (1.04–3.92)	2.36 (1.16–4.81)

Age	Pain interference				

≤50	≤3	178 (79.8)	45 (20.2)	1.0	1.0
≤50	>3	81 (57.0)	61 (43.0)	2.98 (1.87–4.75)	2.68 (1.62–4.43)
>50	≤3	156 (94.0)	10 (6.0)	0.25 (0.12–0.52)	0.29 (0.14–0.60)
>50	>3	33 (67.3)	16 (32.7)	1.92 (0.97–3.79)	2.31 (1.11–4.81)

Note: smoking status, prior abdominal surgery, anxiety and/or depression, and malignancy status were included in adjusted model.

## Additional Points

This study examines pain and healthcare use (HCU) in 590 women in the year prior to gynaecological surgery. One-third reported moderate to severe (>3/10) pain intensity and interference before surgery. Three hundred and sixty women reported 2026 healthcare visits in the year before surgery. The odds of high HCU (≥3 pain-related visits to family doctor or specialist annually, ≥1 to emergency/urgent care) increased in the presence of moderate to severe pain. These findings illustrate the high burden of pain in women awaiting gynaecological surgery and its impact on healthcare use.
